# Supplementary material for: Unraveling the link between neuropathy target esterase NTE/SWS, lysosomal storage diseases, inflammation, abnormal fatty acid metabolism, and leaky brain barrier
Source: eLife. 2024 Apr 25;13:e98020. doi: 10.7554/eLife.98020 (PMC11090517; doi:10.7554/eLife.98020)
Supplement: Supplementary file 1. — a – the ΔCT value is determined by subtracting the average CT value of endogenous control gene(Rpl32) from the average mRNA CT value. b –the calculation of ΔΔCT involves subtraction by the ΔCT calibrator value (ΔCT value in control). c – the range is given for relative levels determined by evaluating the expression: 2–ΔΔCT. AVE ± SEM values are reported from experiments done in at least duplicates. Two-tailed Student’s test was used to test for statistical significance. pa – compared to the relevant control. pb – compared to 15-day-old animals of the same genotype. pc – compared to sws1 mutant of the same age. [file elife-98020-supp1.docx]

### **Supplementary file 1. Relative mRNA levels**

| **Relative *sws* mRNA levels** | | | | | |
| --- | --- | --- | --- | --- | --- |
| ***Genotype*** | ***sws* C_T_** | ***Rpl32* C_T_** | **^a^ΔC_T_** | **^b^ΔΔC_T_** | **^c^Relative *mRNA* levels** |
| *repo>/Oregon R* | 19.5±0.51 | 16.5±0.76 | 3.3±0.27 | 8.9E-17±0.06 | 1.0±0.04 |
| *repo>sws^RNAi^* | 19.7±0.19 | 16.1±0.42 | 3.5±0.28 | 0.3±0.11 | 0.8±0.06  **p^a^= 5.8E-4** |
| *repo>sws* | 17.2±0.24 | 16.2±0.92 | 1.0±0.69 | -2.2±0.43 | 5.0±1.42  **p^a^= 6.7E-4** |
| *nSyb>/Oregon R* | 19.0±0.44 | 15.4±0.56 | 3.5±0.16 | 7.4E-17±0.11 | 1.0±0.08 |
| *nSyb>sws^RNAi^* | 21.1±0.44 | 16.2±0.46 | 4.8±0.21 | 1.4±0.21 | 0.4±0.05  **p^a^= 5.0E-7** |
| *nSyb>sws* | 18.0±0.15 | 15.9±0.61 | 2.2±0.48 | -1.7±0.37 | 2.6±0.71  **p^a^= 4.2E-3** |
| *repo, nSyb>/Oregon R* | 18.4±0.03 | 15.0±0.07 | 3.4±0.03 | -1.5E-16±0.03 | 1.0±0.02 |
| *repo, nSyb>sws^RNAi^* | 19.4±0.03 | 15.2±0.03 | 4.5±0.03 | 0.8±0.03 | 0.6±0.01  **p^a^= 7.9E-6** |
| ***sws* and *moody* mutants show upregulated expression of AMPs** | | | | | |
| ***Genotype*** | ***Attacin A* C_T_** | ***Rpl32* C_T_** | **^a^ΔC_T_** | **^b^ΔΔC_T_** | **^c^Relative *mRNA* levels** |
| *Oregon R* | 21.6±0.70 | 16.2±0.16 | 5.4±0.77 | 3.0E-16±0.18 | 1.0±0.02 |
| *sws^1^* | 19.1±0.68 | 15.7±0.28 | 3.4±0.84 | -2.0±0.14 | 4.2±0.45  **p^a^= 2.2E-3** |
| *white^1118^* | 22.0±0.73 | 15.4±0.22 | 6.6±0.52 | -9.9E-17±0.02 | 1.0±2.1E-4 |
| *moody^ΔC17^* | 18.9±0.74 | 15.1±0.20 | 3.8±0.60 | -2.8±0.11 | 6.9±0.57  **p^a^= 5.1E-4** |
| ***Genotype*** | ***Cecropin A* C_T_** | ***Rpl32* C_T_** | **^a^ΔC_T_** | **^b^ΔΔC_T_** | **^c^Relative *mRNA* levels** |
| *Oregon R* | 22.3±0.96 | 16.1±0.18 | 6.2±1.11 | -9.9E-17±0.02 | 1.0±6.4E-5 |
| *sws^1^* | 20.1±0.90 | 15.8±0.20 | 4.3±1.05 | -1.9±0.07 | 3.8±0.20  **p^a^= 1.6E-4** |
| *white^1118^* | 22.4±1.37 | 16.1±0.53 | 6.3±0.91 | 9.9E-17±0.03 | 1.0±3.4E-4 |
| *moody^ΔC17^* | 20.1±1.48 | 15.9±0.65 | 4.0±0.98 | -2.5±0.08 | 5.6±0.30  **p^a^= 1.0E-4** |
| ***Genotype*** | ***Diptericin* C_T_** | ***Rpl32* C_T_** | **^a^ΔC_T_** | **^b^ΔΔC_T_** | **^c^Relative *mRNA* levels** |
| *Oregon R* | 22.9±0.08 | 16.3±0.15 | 6.6±0.10 | 0.0±0.06 | 1.0±1.0E-3 |
| *sws^1^* | 21.4±0.26 | 16.0±0.22 | 5.4±0.11 | -1.3±0.15 | 2.4±0.19  **p^a^= 1.8E-2** |
| *white^1118^* | 21.4±0.68 | 15.6±0.39 | 5.8±0.51 | 3.0E-16±0.03 | 1.0±2.9E-4 |
| *moody^ΔC17^* | 19.9±0.76 | 15.3±0.53 | 4.6±0.68 | -1.2±0.19 | 2.3±0.33  **p^a^=** **1.7E-2** |
| **Age-dependent increase in AMPs expression is triggered by *sws* loss** | | | | | |
| ***Genotype*** | ***Attacin A* C_T_** | ***Rpl32* C_T_** | **^a^ΔC_T_** | **^b^ΔΔC_T_** | **^c^Relative *mRNA* levels** |
| *Oregon R* 15d | 21.3±0.69 | 14.8±0.21 | 6.4±0.49 | -9.9E-17±0.07 | 1.0± 0.05 |
| *sws^1^* 15d | 19.8±0.30 | 15.2±0.23 | 4.6±0.08 | -1.3±0.04 | 2.5±0.07  **p^a^= 1.6E-13** |
| *sws^1^; moody>sws* 15d | 19.3±0.45 | 15.1±0.25 | 4.2±0.25 | -1.7±0.17 | 3.2±0.4  **p^c^= 9.1E-3** |
| *moody>/Oregon R* 15d | 22.3±0.39 | 14.9±0.20 | 7.4±0.27 | -2.9E-16±0.08 | 1.0±0.06 |
| *moody>sws^RNAi^* 15d | 20.2±0.21 | 15.1±0.37 | 5.1±0.55 | -2.0±0.30 | 4.3±0.86  **p^a^= 1.8E-8** |
| *Oregon R* 30d | 19.6±0.34 | 15.1±0.07 | 4.5±0.38 | -2.3±0.23 | 5.1±0.81  **p^b^=** **6.3E-8** |
| *sws^1^* 30d | 17.1±0.36 | 14.8±0.23 | 2.3±0.14 | -3.6±0.06 | 11.9±0.46  **p^a^= 1.9E-7**  **p^b^=** **9.1E-12** |
| *sws^1^; moody>sws* 30d | 17.9±0.35 | 14.9±0.11 | 3.0±0.39 | -2.9±0.30 | 7.5±1.51  **p^b^= 7.6E-4**  **p^c^=** **7.3E-4** |
| *moody>/Oregon R* 30d | 20.6±0.73 | 14.6±0.39 | 6.0±0.49 | -1.6±0.31 | 3.3±0.71  **p^b^= 3E-7** |
| *moody>sws^RNAi^* 30d | 17.6±0.58 | 15.0±0.38 | 2.7±0.22 | -4.4±0.38 | 23.0±4.96  **p^a^= 4.2E-6**  **p^b^=** **8.1E-6** |
| ***Genotype*** | ***Cecropin A* C_T_** | ***Rpl32* C_T_** | **^a^ΔC_T_** | **^b^ΔΔC_T_** | **^c^Relative *mRNA* levels** |
| *Oregon R* 15d | 22.4±0.05 | 14.8±0.21 | 7.5±0.23 | -2.0E-16±0.05 | 1.0±0.03 |
| *sws^1^* 15d | 21.3±0.04 | 15.4±0.06 | 5.9±0.03 | -1.4±0.15 | 2.7±0.27  **p^a^= 6.6E-9** |
| *sws^1^; moody>sws* 15d | 21.2±0.65 | 15.1±0.17 | 6.1±0.61 | -1.5±0.53 | 3.3±0.95  **p^c^= 0.43** |
| *moody>/Oregon R* 15d | 22.7±0.20 | 14.9±0.20 | 7.8±0.30 | -3.0E-16±0.04 | 1.0±0.03 |
| *moody>sws^RNAi^* 15d | 21.4±0.30 | 15.1±0.37 | 6.3±0.40 | -1.8±0.37 | 3.9±0.84  **p^a^= 1.3E-7** |
| *Oregon R* 30d | 20.5±0.20 | 15.1±0.07 | 5.4±0.16 | -1.9±0.03 | 3.8±0.09  **p^b^= 4.1E-17** |
| *sws^1^* 30d | 17.1±0.42 | 14.7±0.23 | 2.3±0.20 | -5.4±0.39 | 45.7±11.83  **p^a^= 1.1E-4**  **p^b^=** **9E-5** |
| *sws^1^; moody>sws* 30d | 19.9±0.43 | 14.9±0.08 | 5.0±0.450 | -2.5±0.56 | 7.0±2.68  **p^b^= 3.8E-2**  **p^c^=** **1.5E-5** |
| *moody>/Oregon R* 30d | 21.2±0.37 | 15.0±0.35 | 6.3±0.30 | -1.4±0.34 | 2.9±0.85  **p^b^=** **5.6E-5** |
| *moody>sws^RNAi^* 30d | 18.4±0.53 | 15.2±0.39 | 3.1±0.16 | -4.9±0.23 | 29.6±5.56  **p^a^= 1.5E-7**  **p^b^=** **8.9E-7** |
| ***Genotype*** | ***Diptericin* C_T_** | ***Rpl32* C_T_** | **^a^ΔC_T_** | **^b^ΔΔC_T_** | **^c^Relative *mRNA* levels** |
| *Oregon R* 15d | 22.9±0.44 | 14.8±0.21 | 8.1±0.33 | 2.0E-16±0.06 | 1.0±0.04 |
| *sws^1^* 15d | 20.6±0.57 | 15.2±0.22 | 5.5±0.36 | -3.0±0.15 | 7.9±0.83  **p^a^= 1.5E-10** |
| *sws^1^; moody>sws* 15d | 22.1±0.38 | 15.0±0.20 | 7.1±0.53 | -1.3±0.73 | 3.0±1.34  **p^c^= 3.1E-4** |
| *moody>/Oregon R* 15d | 24.1±0.71 | 14.9±0.20 | 9.2±0.66 | -5.9E-17±0.03 | 1.0±0.02 |
| *moody>sws^RNAi^* 15d | 21.6±0.47 | 15.0±0.38 | 6.6±0.71 | -2.5±0.51 | 6.6±1.94  **p^a^= 1.5E-6** |
| *Oregon R* 30d | 21.4±0.94 | 14.9±0.25 | 6.6±1.18 | -1.18±0.89 | 3.0±1.54  **p^b^= 1.5E-2** |
| *sws^1^* 30d | 17.6±0.12 | 14.7±0.23 | 2.9±0.13 | -4.9±0.17 | 30.3±3.62  **p^a^= 2.9E-7**  **p^b^=** **1.0E-6** |
| *sws^1^; moody>sws* 30d | 20.3±0.51 | 14.9±0.11 | 5.36±0.55 | -3.1±0.34 | 8.7±1.99  **p^b^= 2.1E-3**  **p^c^=** **3.9E-6** |
| *moody>/Oregon R* 30d | 22.8±0.29 | 14.5±0.41 | 8.3±0.15 | -1.94±0.38 | 4.2±0.9  **p^b^= 6.4E-8** |
| *moody>sws^RNAi^* 30d | 20.2±0.82 | 15.6±0.16 | 4.6±0.97 | -4.0±0.13 | 15.8±1.66 **p^a^= 3.0E-8**  **p^b^=** **5.9E-5** |
| ***sws* downregulation in glial cells after the BBB formation leads to the increased inflammatory response** | | | | | |
| ***Genotype*** | ***Attacin A* C_T_** | ***Rpl32* C_T_** | **^a^ΔC_T_** | **^b^ΔΔC_T_** | **^c^Relative *mRNA* levels** |
| *tub-Gal80^ts^; repo>/Oregon R* 7d | 21.3±0.86 | 14.8±0.11 | 6.6±0.91 | 4.0E-16±0.024 | 1.0±1.7E-4 |
| *tub-Gal80ts; repo>sws^RNAi^* 7d | 21.5±0.58 | 14.6±0.18 | 6.9±0.65 | 0.3±0.53 | 1.0±0.36 |
| *tub-Gal80^ts^; repo>/Oregon R* 15d | 20.7±0.65 | 14.8±0.06 | 5.9±0.70 | -0.7±0.64 | 2.1±1.01 |
| *tub-Gal80ts; repo>sws^RNAi^* 15d | 19.1±0.81 | 14.7±0.20 | 4.4±0.71 | -3.2±0.32 | 9.3±2.54 |
| ***Genotype*** | ***Cecropin A* C_T_** | ***Rpl32* C_T_** | **^a^ΔC_T_** | **^b^ΔΔC_T_** | **^c^Relative *mRNA* levels** |
| *tub-Gal80^ts^; repo>/Oregon R* 7d | 22.2±1.13 | 14.8±0.11 | 7.4±1.19 | 9.9E-17±0.03 | 1.0±3.3E-3 |
| *tub-Gal80ts; repo>sws^RNAi^* 7d | 21.6±0.98 | 14.6±0.18 | 7.0±1.10 | -0.4±1.31 | 3.7±3.2 |
| *tub-Gal80^ts^; repo>/Oregon R* 15d | 23.0±0.14 | 14.8±0.08 | 8.2±0.19 | -0.6±0.12 | 1.5±0.09 |
| *tub-Gal80ts; repo>sws^RNAi^* 15d | 19.7±0.61 | 14.7±0.20 | 5.0±0.51 | -3.8±0.42 | 14.4±5.18 |
| ***Genotype*** | ***Diptericin* C_T_** | ***Rpl32* C_T_** | **^a^ΔC_T_** | **^b^ΔΔC_T_** | **^c^Relative *mRNA* levels** |
| *tub-Gal80^ts^; repo>/Oregon R* 7d | 23.2±0.91 | 14.8±0.11 | 8.5±0.91 | 0.0±0.08 | 1.0±4.2E-3 |
| *tub-Gal80ts; repo>sws^RNAi^* 7d | 22.2±0.75 | 14.6±0.18 | 7.6±0.78 | -0.6±0.21 | 1.6±0.23 |
| *tub-Gal80^ts^; repo>/Oregon R* 15d | 23.2±1.08 | 14.8±0.08 | 8.4±1.15 | 0.3±0.32 | 0.9±0.24 |
| *tub-Gal80ts; repo>sws^RNAi^* 15d | 19.6±0.83 | 14.5±0.17 | 5.1±0.90 | -2.8±1.61 | 24.4±13.95 |

^a^ - the ΔCT value is determined by subtracting the average CT value of endogenous control gene (Rpl32) from the average mRNA CT value.

^b^ - the calculation of ΔΔCT involves subtraction by the ΔCT calibrator value (ΔCT value in control).

^c^ - the range is given for relative levels determined by evaluating the expression: 2-ΔΔCT.

AVE±SEM values are reported from experiments done in at least duplicates. Two-tailed Student’s test was used to test for statistical significance.

p^a^ – compared to the relevant control

p^b^ – compared to 15-day-old animals of the same genotype

p^c^ – compared to *sws^1^* mutant of the same age
