# Supplementary material for: Unraveling the link between neuropathy target esterase NTE/SWS, lysosomal storage diseases, inflammation, abnormal fatty acid metabolism, and leaky brain barrier
Source: eLife. 2024 Apr 25;13:e98020. doi: 10.7554/eLife.98020 (PMC11090517; doi:10.7554/eLife.98020)
Supplement: Supplementary file 2. — a – compared to control (OR x w1118). b – compared to Gal4-driver x OR. c – compared to Gal4-driver x UAS-swsRNAi. The values are reported from experiments done in triplicates. For statistical analyses of the observed phenotypes, two-way tables and chi-squared test were used. [file elife-98020-supp2.docx]

### **Supplementary file 2. NTE/SWS expression in the surface glia is important for the integrity of Drosophila BBB**

| *Genotype* | Phenotypes | | | P-value | Number of brain hemi-spheres analyzed |
| --- | --- | --- | --- | --- | --- |
|  | **No lesions** | **Lesions** | **Lesions + membrane clusters** |  |  |
| *Control*  *OR x w^1118^* | 100% | 0% | 0% |  | 96 |
| *sws^1^* | 14% | 11% | 75% | ^a^p = 9.9E-31 | 85 |
| *sws^1^/sws^4^* | 13% | 28% | 59% | ^a^p = 3.3E-29 | 69 |
| *repo, nSyb>sws^RNAi^* | 7% | 24% | 69% | ^a^p = 1.5E-31 | 62 |
| *repo>/Oregon R* | 99% | 1% | 0% | ^a^p = 0.27 | 80 |
| *repo>sws^RNAi^* | 1% | 13% | 86% | ^b^p = 1.2E-31 | 70 |
| *sws^1^; repo>sws* | 100% | 0% | 0% | ^b^p = 0.46  ^c^p = 2.3E-24 | 43 |
| *sws^1^; repo>hNTE* | 100% | 0% | 0% | ^b^p = 1  ^c^p = 1.6E-28 | 62 |
| *moody>/Oregon R* | 100% | 0% | 0% | ^a^p = 1 | 67 |
| *moody>sws^RNAi^* | 15% | 15% | 70% | ^b^p = 1.1E-22 | 72 |
| *sws^1^; moody>sws* | 98% | 2% | 0% | ^b^p = 0.3  ^c^p = 4.3E-21 | 63 |
| *sws^1^; moody>hNTE* | 100% | 0% | 0% | ^b^p = 1  ^c^p = 1.9E-21 | 61 |
| *Gli>/Oregon R* | 100% | 0% | 0% | ^a^p = 1 | 58 |
| *Gli>sws^RNAi^* | 8% | 12% | 80% | ^b^p = 2.4E-22 | 60 |
| *sws^1^; Gli>sws* | 98% | 0% | 2% | ^b^p = 0.34  ^c^p = 5.5E-23 | 65 |
| *sws^1^; Gli>hNTE* | 100% | 0% | 0% | ^b^p = 1  ^c^p = 8.9E-23 | 60 |
| *moody* *^ΔC17^* | 17% | 23% | 60% | ^a^p = 1.2E-32 | 118 |
| *moody>moody^RNAi^* | 4% | 0% | 96% | ^a^p = 1.5E-27 | 28 |

^a^ – compared to control (*OR x w^1118^*)

^b^ – compared to *Gal4-driver x OR*

^c^ – compared to *Gal4-driver x UAS-sws^RNAi^*

The values are reported from experiments done in triplicates. For statistical analyses of the observed phenotypes, two-way tables and χ^2^-test were used.
