# Supplementary material for: Unraveling the link between neuropathy target esterase NTE/SWS, lysosomal storage diseases, inflammation, abnormal fatty acid metabolism, and leaky brain barrier
Source: eLife. 2024 Apr 25;13:e98020. doi: 10.7554/eLife.98020 (PMC11090517; doi:10.7554/eLife.98020)
Supplement: Supplementary file 3. — a – compared to Gal4-driver x OR animals of the same age. b – compared to 1-day-old animals of the same genotype. The values are reported from experiments done in triplicates. For statistical analyses of the observed phenotypes, two-way tables and chi-squared test were used. [file elife-98020-supp3.docx]

### **Supplementary file 3. NTE/SWS deficit in the surface glia results in the accumulation of Rab7- and CathepsinL-positive structures**

| *Genotype* | Rab7 accumulation in the surface glia | | P-value | Number of brain hemispheres analyzed |
| --- | --- | --- | --- | --- |
|  | **No** | **Yes** |  |  |
| *moody>/Oregon R* 1d | 96% | 4% |  | 71 |
| *moody>sws^RNAi^* 1d | 58% | 42% | ^a^p = 9.3E-8 | 77 |
| *moody>/Oregon R* 15d | 95% | 5% | ^b^p = 0.82 | 59 |
| *sws^1^; moody>CD8::GFP* 15d | 18% | 82% | ^a^p = 2E-15 | 44 |
| *moody>sws^RNAi^* 15d | 31% | 69% | ^a^p = 3.4E-13  ^b^p = 1E-3 | 62 |
| *Genotype* | **CathepsinL accumulation in the surface glia** | | **P-value** | **Number of brain hemispheres analyzed** |
|  | **No** | **Yes** |  |  |
| *moody>/Oregon R* 1d | 90% | 10% |  | 80 |
| *moody>sws^RNAi^* 1d | 59% | 41% | ^a^p = 7.6E-6 | 81 |
| *moody>/Oregon R* 15d | 89% | 11% | ^b^p = 0.8 | 62 |
| *sws^1^; moody>CD8::GFP* 15d | 43% | 57% | ^a^p = 2.3E-7 | 51 |
| *moody>sws^RNAi^* 15d | 37% | 63% | ^a^p = 1E-8  ^b^p = 0.013 | 49 |

^a^ – compared to *Gal4-driver x OR* animals of the same age

^b^ – compared to 1-day-old animals of the same genotype

The values are reported from experiments done in triplicates. For statistical analyses of the observed phenotypes, two-way tables and χ^2^-test were used.
