# Supplementary material for: Unraveling the link between neuropathy target esterase NTE/SWS, lysosomal storage diseases, inflammation, abnormal fatty acid metabolism, and leaky brain barrier
Source: eLife. 2024 Apr 25;13:e98020. doi: 10.7554/eLife.98020 (PMC11090517; doi:10.7554/eLife.98020)
Supplement: Supplementary file 4. — a – compared to control (OR x w1118). b – compared to Gal4-driver x OR. c –compared to Gal4-driver x UAS-swsRNAi. The values are reported from experiments done in triplicates. For statistical analyses of the observed phenotypes, two-way tables and chi-squared test were used. [file elife-98020-supp4.docx]

### **Supplementary file 4. NTE/SWS deficit in the surface glia results in permeable BBB**

| *Genotype* | BBB permeability | | P-value | Number of brain hemispheres analyzed |
| --- | --- | --- | --- | --- |
|  | **Normal** | **Permeable** |  |  |
| *Control* (*Or x w^1118^*) | 75% | 25% |  | 96 |
| *sws^1^* | 12% | 88% | ^a^p = 1.4E-18 | 94 |
| *sws^1^/sws^4^* | 2% | 98% | ^a^p = 7.7E-18 | 54 |
| *repo, nSyb>sws^RNAi^* | 12% | 88% | ^a^p=1.01E-13 | 56 |
| *repo>/Oregon R* | 66% | 34% | ^a^p = 0.23 | 59 |
| *repo>sws^RNAi^* | 9% | 91% | ^a^p = 1.8E-14  ^b^p = 8.7E-10 | 53 |
| *sws^1^; repo>sws* | 18% | 82% | ^b^p = 4E-4  ^c^p = 0.21 | 17 |
| *sws^1^; repo>hNTE* | 36% | 64% | ^b^p = 2.8E-3  ^c^p = 1.4E-3 | 44 |
| *moody>/Oregon R* | 66% | 34% | ^a^p = 0.17 | 91 |
| *moody>sws^RNAi^* | 9% | 91% | ^a^p = 4.1E-16  ^b^p = 2.4E-12 | 64 |
| *sws^1^; moody>sws* | 22% | 78% | ^b^p = 1.9E-8  ^c^p = 0.046 | 73 |
| *sws^1^; moody>hNTE* | 16% | 84% | ^b^p = 1.4E-8  ^c^p = 0.29 | 50 |
| *Gli>/Oregon R* | 65% | 35% | ^a^p = 0.15 | 71 |
| *Gli>sws^RNAi^* | 8% | 92% | ^a^p = 2.1E-16  ^b^p = 1.9E-11 | 62 |
| *sws^1^; Gli>sws* | 25% | 75% | ^b^p = 3.9E-6  ^c^p = 0.01 | 61 |
| *sws^1^; Gli>hNTE* | 26% | 74% | ^b^p = 2.6E-5  ^c^p = 0.01 | 50 |

^a^ – compared to control (*OR x w^1118^*)

^b^ – compared to *Gal4-driver x OR*

^c^ – compared to *Gal4-driver x UAS-sws^RNAi^*

The values are reported from experiments done in triplicates. For statistical analyses of the observed phenotypes, two-way tables and χ^2^-test were used.
