# Supplementary material for: Unraveling the link between neuropathy target esterase NTE/SWS, lysosomal storage diseases, inflammation, abnormal fatty acid metabolism, and leaky brain barrier
Source: eLife. 2024 Apr 25;13:e98020. doi: 10.7554/eLife.98020 (PMC11090517; doi:10.7554/eLife.98020)
Supplement: Supplementary file 5. — a – compared to sws1 (no drug treatment)b – compared to moody ΔC17 (no drug treatment). The values are reported from experiments done in triplicates. For statistical analyses of the observed phenotypes, two-way tables and chi-squared test were used. [file elife-98020-supp5.docx]

### **Supplementary file 5. The effect of treatment with different anti-inflammatory substances and stress suppressors on the frequency of the surface glia phenotype in *sws* and *moody* mutants**

| *Genotype* + Drug | % of brain hemispheres with CoraC phenotype | Delta % after treatment | P-value | Number of brain hemispheres analyzed |
| --- | --- | --- | --- | --- |
| *sws^1^* | 75% |  |  | 85 |
| *sws^1^ + 5% Glucose* | 74% | -1% | ^a^p = 0.9 | 94 |
| *sws^1^ +* *Tauroursodeoxycholic acid (TUDCA) 0.015M* | 81% | 6% | ^a^p = 0.31 | 102 |
| *sws^1^ + Sodium 4-Phenylbutyrate (4-PBA) 0.02M* | 70% | -6% | ^a^p = 0.37 | 139 |
| *sws^1^* *+ Valsartan 0.02M* | 66% | -10% | ^a^p = 0.15 | 114 |
| *sws^1^* *+ Fenofibrate 0.02M* | 78% | 2% | ^a^p = 0.71 | 116 |
| *sws^1^* *+* *Sodium Salicylate 0.03M* | 53% | -22% | ^a^p = 1E-3 | 144 |
| *sws^1^* *+* *Rapamycin (Rap) 0.0001M* | 62% | -13% | ^a^p = 0.05 | 132 |
| *sws^1^* *+* *Deferoxamine mesylate salt (DFO) 0.0005M* | 64% | -11% | ^a^p = 0.08 | 128 |
| *sws^1^* *+* *Liproxstatin-1 0.004M* | 70% | -5% | ^a^p = 0.45 | 101 |
| *sws^1^* *+* *Sphingosine (BS) 0.003M* | 64% | -11% | ^a^p = 0.14 | 73 |
| *sws^1^ + 5%Glucose* | 79% |  |  | 134 |
| *sws^1^ + Sodium Sal. 0.03M* | 49% | -30% | ^a^p = 4E-7 | 130 |
| *sws^1^ + Rapamycin 0.0001M* | 62% | -17% | ^a^p = 1.8E-3 | 133 |
| *moody ^ΔC17^ + 5%Glucose* | 60% |  |  | 118 |
| *moody ^ΔC17^ + Sodium Sal. 0.03M* | 46% | -14% | ^b^p = 0.02 | 156 |
| *moody ^ΔC17^ + Rapamycin 0.0001M* | 39% | -21% | ^b^p = 2E-3 | 104 |

^a^ – compared to *sws^1^* (no drug treatment)

^b^ – compared to *moody ^ΔC17^* (no drug treatment)

The values are reported from experiments done in triplicates. For statistical analyses of the observed phenotypes, two-way tables and χ^2^-test were used.
