# Supplementary material for: Unraveling the link between neuropathy target esterase NTE/SWS, lysosomal storage diseases, inflammation, abnormal fatty acid metabolism, and leaky brain barrier
Source: eLife. 2024 Apr 25;13:e98020. doi: 10.7554/eLife.98020 (PMC11090517; doi:10.7554/eLife.98020)
Supplement: Supplementary file 6. — (a) sws and moody mutants show upregulated levels of free fatty acids (FFAs). For statistical analyses one-way ANOVA test was used. C14:1–9-cis-Tetradecenoic acid. C16:0 – Palmitic acid. C16:1 – Palmitoleic acid. C18:0 – Stearic acid. C18:1 – Oleic acid. C18:2 – Linoleic acid. C18:3 – α- and γ-Linolenic acid. C20:0 – Eicosanoic acid. C20:4 – Arachidonic acid. C20:5 – Eicosapentaenoic acid. (b) sws and moody mutants show upregulated levels of free fatty acids (FFAs). Summary of the ions monitored in the selected-ion monitoring (SIM) modeSIM#1 (12.00–14.50 min): m/z 197.4, 199.4, 225.4, 227.4, 253.4, 255.4, 267.4, 269.4. SIM#2 (14.50–15.00 min): m/z 281.4, 283.4, 279.4, 295.4, 297.4. SIM#3 (15.00–17.00 min): m/z 301.4, 303.4, 309.4, 311.4, 325.4, 337.4, 339.4, 365.4, 367.4. [file elife-98020-supp6.docx]

### **Supplementary file 6a. *sws* and *moody* mutants show upregulated levels of free fatty acids (FFAs)**

| *Genotype* | C14:1 | | C16:0 | | C16:1 | | C18:0 | C18:1 | C18:2 | C18:3 | C20:0 | C20:4 | C20:5 |
| --- | --- | --- | --- | --- | --- | --- | --- | --- | --- | --- | --- | --- | --- |
|  | *FA/IS by weight (Average)* | | | | | | | | | | | | |
| *Control Oregon R* | 1.7x10^2^ | | 8.5x10^2^ | | 1.1x10^3^ | | 3.2x10^2^ | 7.8x10^2^ | 7.2x10^2^ | 8.0x10^2^ | 1.9 | 6.3x10^-3^ | 6.4x10^-3^ |
| *sws^1^* | 3.1x10^2^ | | 2.4x10^3^ | | 2.7x10^3^ | | 6.3x10^2^ | 1.9x10^3^ | 1.8x10^3^ | 1.4x10^3^ | 6.8 | 1.7x10^-2^ | 9.4x10^-3^ |
| P-value | p=1E-6 | | | | | | | | | | | | |
| *Genotype* | **C14:1** | **C16:0** | | **C16:1** | | **C18:0** | | **C18:1** | **C18:2** | **C18:3** | **C20:0** | **C20:4** | **C20:5** |
|  | *FA/IS by weight (Average)* | | | | | | | | | | | | |
| *Control white^1118^* | 2.3x10^2^ | | 1.1x10^3^ | | 1.5x10^3^ | | 3.7x10^2^ | 1.1x10^3^ | 9.5x10^2^ | 1.1x10^3^ | 2.1 | 8.6x10^-3^ | 5.8x10^-3^ |
| *moody ^ΔC17^* | 2.7x10^2^ | | 2.0x10^3^ | | 2.9x10^3^ | | 6.1x10^2^ | 2.2x10^3^ | 2.0x10^3^ | 2.2x10^3^ | 4.1 | 2.0x10^-2^ | 1.3x10^-2^ |
| P-value | p=6.7E-9 | | | | | | | | | | | | |

For statistical analyses one-way ANOVA test was used.

C14:1 - 9-cis-Tetradecenoic acid

C16:0 - Palmitic acid

C16:1 - Palmitoleic acid

C18:0 - Stearic acid

C18:1 - Oleic acid

C18:2 - Linoleic acid

C18:3 - α- and γ-Linolenic acid

C20:0 - Eicosanoic acid

C20:4 - Arachidonic acid

C20:5 - Eicosapentaenoic acid

Summary of the ions monitored in the SIM mode

SIM#1 (12.00-14.50 min):

*m/z* 197.4, 199.4, 225.4, 227.4, 253.4, 255.4, 267.4, 269.4

SIM#2 (14.50-15.00 min):

*m/z* 281.4, 283.4, 279.4, 295.4, 297.4

SIM#3 (15.00-17.00 min):

*m/z* 301.4, 303.4, 309.4, 311.4, 325.4, 337.4, 339.4, 365.4, 367.4

### **Supplementary file 6b. FFAs analyzed by GC-MS after derivatization to their pentafluorobenzyl esters**

| **Fatty acid** | **Acronym** | **MW**  **(Da)** | ***t*_R_**  **(min)** | **SIM**  **(*m/z*)** |
| --- | --- | --- | --- | --- |
|  |  |  |  |  |
| Dodecanoic acid (lauric acid) | C12:0 | 200.3 | 12.62 | 199 |
| (5Z)-Dodecenoic acid | C12:1 | 198.3 | 12.65 | 197 |
| Tetradecanoic acid (myristic acid) | C14:0 | 228.4 | 13.44 | 227 |
| 9-cis-Tetradecenoic acid | C14:1 | 226.4 | 13.52 | 225 |
| Hexadecanoic acid (palmitic acid) | C16:0 | 256.4 | 14.07 | 255 |
| 9-cis-Hexadecenoic acid | C16:1 | 254.4 | 14.10 | 253 |
| Heptadecanoic acid | C17:0 | 270.4 | 14.31 | 269 |
| Heptadecenoic acid | 10c-C17:1 | 268.4 | 14.39 | 267 |
| Octadecanoic acid (stearic acid) | C18:0 | 284.5 | 14.58 | 283 |
| 9-cis-Octadecenoic acid (oleic acid) | C18:1 | 282.5 | 14.62 | 281 |
| 9-trans-Octadecenoic acid | 9t-C18:1 | 282.5 | 14.62 | 281 |
| 11-trans-Octadecenoic acid | 1t-C18:1 | 282.5 | 14.64 | 281 |
| 6-cis-Octadecenoic acid | 6c-C18:1 | 282.5 | 14.60 | 281 |
| Linoleic acid | C18:2 | 280.5 | 14.68 | 279 |
| alpha-Linolenic acid | C18:3 | 278.4 | 14.76 | 277 |
| gamma-Linolenic acid | C18:3 | 278.4 | 14.68 | 277 |
| Nonadecanoic acid | C19:0 | 298.5 | 14.83 | 297 |
| **INTERNAL STANDARD** | **8-cp-C18:0** | **296.5** | **14.81** | **295** |
| Eicosanoic acid | C20:0 | 312.5 | 15.05 | 311 |
| 11-cis-Eicosenoic acid | 11c-C20:1 | 310.5 | 15.08 | 309 |
| Arachidonic acid (AA) | C20:4 | 304.5 | 15.13 | 303 |
| Eicosapentanoic acid (EPA) | C20:5 | 302.5 | 15.22 | 301 |
| Heneisosanoic acid | C21:0 | 326.6 | 15.26 | 325 |
| Docosanoic acid | C22:0 | 340.6 | 15.46 | 339 |
| Erucic acid | 13c-C22:1 | 338.6 | 15.52 | 337 |
| Tetracosanoic acid | C24:0 | 368.6 | 15.85 | 367 |
| 15-cis-Tetracosenoic acid | 15c-C24:1 | 366.6 | 15.99 | 365 |

### **Supplementary file 6c. Mean peak area ratio and its coefficient of variation (CV) of the listed free fatty acids (FFA) to the internal standard (IS) in the diluted (1:10, v/v) control standard sample (10 nmol arachidonic acid, 10 nmol internal standard) analyzed in duplicate as pentafluorobenzyl esters**

| **Fatty acid** | **Acronym** | **FFA/IS** | **CV (%)** |
| --- | --- | --- | --- |
| Dodecanoic acid (lauric acid) | C12:0 | 4.3 E-03 | 13.8 |
| (5Z)-Dodecenoic acid | C12:1 | 6.0 E-04 | 15.1 |
| Tetradecanoic acid (myristic acid) | C14:0 | 5.3 E-03 | 10.7 |
| 9-cis-Tetradecenoic acid | C14:1 | 6.0 E-04 | 1.3 |
| Hexadecanoic acid (palmitic acid) | C16:0 | **2.6 E-02** | 7.7 |
| 9-cis-Hexadecenoic acid | C16:1 | 2.6 E-03 | 1.0 |
| Heptadecanoic acid | C17:0 | 6.0 E-04 | 1.3 |
| Heptadecenoic acid | 10c-C17:1 | 2.1 E-04 | 3.8 |
| Octadecanoic acid (stearic acid) | C18:0 | 6.6 E-03 | 11.1 |
| 9-cis-Octadecenoic acid (oleic acid) | C18:1 | 2.4 E-03 | 4.4 |
| 9-trans-Octadecenoic acid | 9t-C18:1 | not found | |
| 11-trans-Octadecenoic acid | 1t-C18:1 | 2.3 E-03 | 2.0 |
| 6-cis-Octadecenoic acid | 6c-C18:1 | 2.3 E-03 | 2.0 |
| Linoleic acid | C18:2 | 6.1 E-04 | 2.7 |
| alpha-Linolenic acid | C18:3 | 5.7 E-05 | 16.4 |
| gamma-Linolenic acid | C18:3 | 5.6 E-05 | 15.7 |
| Nonadecanoic acid | C19:0 | **2.7 E-02** | 1.1 |
| **INTERNAL STANDARD** | **8-cp-C18:0** | **1:1** | **n.a.** |
| Eicosanoic acid | C20:0 | 6.1 E-05 | 16.0 |
| 11-cis-Eicosenoic acid | 11c-C20:1 | 5.6 E-05 | 38.7 |
| Arachidonic acid (AA) | C20:4 | **9.8 E-02** | 0.8 |
| Eicosapentanoic acid (EPA) | C20:5 | 5.2 E-05 | 21.5 |
| Heneisosanoic acid | C21:0 | 2.0 E-05 | 24.8 |
| Docosanoic acid | C22:0 | 1.9 E-05 | 13.1 |
| Erucic acid | 13c-C22:1 | 3.6 E-05 | 3.6 |
| Tetracosanoic acid | C24:0 | 4.9 E-06 | 27.6 |
| 15-cis-Tetracisenoic acid | 15c-C24:1 | not found | |
